# Deep feature engineering for accurate sperm morphology classification using CBAM-enhanced ResNet50

**DOI:** 10.1371/journal.pone.0330914

**Published:** 2025-09-10

**Authors:** Şafak Kılıç

**Affiliations:** 1 School of Computer Science, CHART Laboratory, University of Nottingham, Nottingham, United Kingdom; 2 Faculty of Engineering, Architecture and Design, Department of Software Engineering, Kayseri University, Kayseri, Turkey; Najran University College of Computer Science and Information Systems, SAUDI ARABIA

## Abstract

**Background and objective:**

Male fertility assessment through sperm morphology analysis remains a critical component of reproductive health evaluation, as abnormal sperm morphology is strongly correlated with reduced fertility rates and poor assisted reproductive technology outcomes. Traditional manual analysis performed by embryologists is time-intensive, subjective, and prone to significant inter-observer variability, with studies reporting up to 40% disagreement between expert evaluators. This research presents a novel deep learning framework combining Convolutional Block Attention Module (CBAM) with ResNet50 architecture and advanced deep feature engineering (DFE) techniques for automated, objective sperm morphology classification.

**Materials and methods:**

We propose a hybrid architecture integrating ResNet50 backbone with CBAM attention mechanisms, enhanced by a comprehensive deep feature engineering pipeline. The framework incorporates multiple feature extraction layers (CBAM, GAP, GMP, pre-final) combined with 10 distinct feature selection methods including Principal Component Analysis (PCA), Chi-square test, Random Forest importance, variance thresholding, and their intersections. Classification is performed using Support Vector Machines with RBF/Linear kernels and k-Nearest Neighbors algorithms. The model was rigorously evaluated on two benchmark datasets: SMIDS (3000 images, 3-class) and HuSHeM (216 images, 4-class) using 5-fold cross-validation.

**Results:**

The proposed framework achieved exceptional performance with test accuracies of 96.08 ± 1.2% on SMIDS dataset and 96.77 ± 0.8% on HuSHeM dataset using deep feature engineering, representing significant improvements of 8.08% and 10.41% respectively over baseline CNN performance. McNemar’s test confirmed statistical significance ($\chi^{2}=24.31,p<0.001$). The best configuration (GAP + PCA + SVM RBF) demonstrated superior performance compared to existing state-of-the-art approaches, including recent Vision Transformer and ensemble methods.

**Conclusions and clinical impact:**

This research demonstrates the effectiveness of attention-based deep learning combined with sophisticated feature engineering for sperm morphology analysis. The proposed framework achieves state-of-the-art performance while providing clinically interpretable results through Grad-CAM attention visualization. Clinical implications include: (1) standardized, objective fertility assessment reducing diagnostic variability, (2) significant time savings for embryologists (from 30–45 minutes to <1 minute per sample), (3) improved reproducibility across laboratories, and (4) potential for real-time analysis during assisted reproductive procedures, ultimately enhancing patient care and treatment outcomes in reproductive medicine.

## Author summary

I am Şafak Kılıç, currently a postdoctoral researcher at the CHART Laboratory, School of Computer Science, University of Nottingham. I received my B.Sc. degree in Computer Education and Instructional Technology from Fırat University in 2011, an M.Sc. degree in Computer Science from the University of Brighton in 2015, and a Ph.D. degree in Computer Engineering from Ankara University in 2021. I am also serving as an Assistant Professor in the Department of Software Engineering at Kayseri University. My research focuses on deep learning, medical image analysis, and computer vision, with particular emphasis on developing accurate and efficient models for biomedical applications, including automated sperm morphology classification.

## Introduction

Sperm morphology refers to the size, shape, and structural characteristics of sperm cells, including head shape, acrosome integrity, neck structure, and tail configuration. According to World Health Organization (WHO) guidelines, normal sperm morphology is characterized by an oval head (length: 4.0–5.5 *μ*m, width: 2.5–3.5 *μ*m), intact acrosome covering 40–70% of the head, and a single, uniform tail [1].

Sperm morphology evaluation is a critical component of male fertility diagnostics, as abnormalities in sperm shape are associated with reduced fertility and poor assisted reproduction outcomes [2]. In routine practice, however, morphology assessment is typically performed manually by trained embryologists following World Health Organisation (WHO) guidelines. Manual sperm morphology assessment suffers from key limitations, including high inter-observer variability (up to 40% CV), lengthy evaluation times (30–45 minutes per sample), inconsistent standards across laboratories, and the need for expert training. Reported kappa values as low as 0.05–0.15 further highlight substantial diagnostic disagreement, even among trained technicians [3]. Automated classification overcomes these barriers by offering objective and reproducible assessments with rapid processing times, consistent inter-lab performance, and reduced dependency on expert personnel. This manual process is labor-intensive and highly subjective, leading to significant observer variability and inconsistency in results [4,5]. For instance, laboratories often must examine at least 200 sperm per sample to obtain a reliable morphology assessment, a tedious task prone to human error. While computer-aided semen analysis (CASA) systems can objectively measure parameters like concentration and motility, they remain unreliable for morphology evaluation [6]. These limitations underscore the need for automated sperm morphology classification methods that can improve accuracy, objectivity, and throughput in clinical settings.

Recent advances in computer vision and artificial intelligence have led to the development of automated sperm morphology analysis techniques. In particular, deep learning via convolutional neural networks (CNNs) has shown remarkable promise for image-based classification tasks in reproductive medicine [7]. Several studies have reported that CNN-based models can achieve expert-level or better performance in classifying normal vs. abnormal sperm forms, thereby reducing reliance on subjective human judgement. For example, Spencer et al. (2022) employed a stacked ensemble of CNNs (combining VGG16, ResNet-34, DenseNet, etc.) to classify human sperm head morphology, attaining a classification accuracy of up to ∼98.2% on the HuSHeM dataset [2]. Similarly, Keller et al. (2024) demonstrated high precision (F1-scores ≈97–99%) in boar sperm morphological classification using deep CNN models [6]. These successes illustrate the potential of deep learning to standardize sperm morphology assessment and reduce technician variability.

Building on recent developments, attention mechanisms and classical feature engineering are integrated within a deep learning architecture to enhance sperm morphology classification performance. Deep feature engineering (DFE) represents an advanced machine learning paradigm that combines the representational power of deep neural networks with classical feature selection and machine learning methods. Unlike end-to-end deep learning approaches, DFE extracts high-dimensional feature representations from intermediate layers of pre-trained networks, applies dimensionality reduction and feature selection techniques, and employs shallow classifiers for final prediction [8]. This hybrid approach enables automatic discovery of meaningful representations while maintaining the interpretability and efficiency benefits of traditional machine learning methods, making it particularly suitable for medical imaging applications where both accuracy and explainability are crucial. Two state-of-the-art CNN architectures, **ResNet50** and **Xception**, are employed as backbone feature extractors and further enhanced using the **Convolutional Block Attention Module** (CBAM). CBAM is a lightweight attention module that sequentially applies channel-wise and spatial attention to intermediate feature maps [9], enabling the network to focus on the most relevant sperm features (e.g., head shape, acrosome size, tail defects) while suppressing background or noise. The integration of CBAM into ResNet50 and Xception aims to enhance the representational capacity of extracted features, particularly for capturing subtle morphological differences between normal and teratozoospermic sperm. Beyond end-to-end CNN classification, performance is further improved through a deep feature engineering (DFE) strategy: **principal component analysis** (PCA) is employed to reduce noise and dimensionality in the deep feature space, followed by a **support vector machine** (SVM) classifier trained on the resulting compact feature set. Such hybrid CNN+DFE approaches have been shown to be effective in other domains, yielding higher accuracies than CNNs alone [10]. For instance, the combination of deep CNN features with an SVM classifier has achieved impressive results (e.g., 95–96% accuracy) in challenging image classification tasks [10]. In our experiments, a ResNet50/Xception model with CBAM achieved a base accuracy of approximately 88% in classifying sperm morphology. Applying PCA to the deep feature embeddings and subsequently training an SVM led to a classification accuracy of **96.08%**, representing a substantial improvement of approximately 8 percentage points. This result underscores the effective synergy between modern attention-augmented deep learning architectures and classical machine learning techniques for feature optimization. Overall, our proposed approach addresses the key limitations of manual sperm morphology analysis by offering an automated solution that is accurate, consistent, and efficient, which could be highly valuable for both clinical diagnostics and reproductive research.

### Literature review

Traditional computer vision methods for sperm morphology analysis, while pioneering in their time, suffer from fundamental limitations that restrict their clinical applicability. The approach by Ilhan et al. (2020a), which utilized wavelet denoising and directional masking followed by handcrafted feature extraction, demonstrated modest improvements of 10% on HuSHeM and 5% on SMIDS datasets. However, this method’s reliance on manually designed features limits its ability to capture subtle morphological variations that may be clinically significant [11]. The computational expense of multi-stage preprocessing and the need for extensive parameter tuning for different imaging conditions further restrict its practical deployment in clinical settings where processing speed and consistency are paramount.

The transition to deep learning approaches marked a significant advancement in automated sperm analysis, yet early implementations revealed important limitations. MobileNet-based approaches, such as the work by Ilhan et al. (2020b), achieved 87% accuracy on SMIDS while offering computational efficiency suitable for mobile deployment[12]. However, the limited representational capacity of lightweight architectures constrains their ability to learn complex morphological patterns, particularly for subtle abnormalities that require fine-grained feature discrimination. The tendency toward overfitting on small medical datasets and the lack of attention mechanisms to focus on morphologically relevant regions further limit their clinical effectiveness.

Ensemble methods, exemplified by Spencer et al. (2022), have achieved the highest performance levels to date, reaching 95.2% accuracy on HuSHeM through stacked generalization of multiple CNN architectures [2]. While these approaches benefit from combining diverse model strengths and achieving robust predictions, they suffer from significant computational overhead that makes real-time clinical deployment challenging. The complexity of training multiple models and combining their outputs creates practical barriers for implementation in resource-constrained clinical environments. Additionally, the black-box nature of ensemble predictions limits interpretability, which is pivotal for clinical decision-making and regulatory compliance in medical applications.

Recent attention-based approaches, including Vision Transformers and Swin Transformer implementations, have introduced sophisticated attention mechanisms to medical image analysis. Mahali et al. (2023) achieved 94.6% accuracy on HuSHeM using a dual architecture fusion approach with autoencoder enhancement [13]. However, these methods typically require substantial computational resources and extensive hyperparameter optimisation, making them less suitable for widespread clinical deployment. The attention mechanisms, while theoretically appealing, often struggle to learn meaningful patterns on small medical datasets and may focus on image artefacts rather than clinically relevant morphological features.

Earlier works in sperm morphology analysis combined classical image processing with machine learning. **Ilhan et al. (2020a)** introduced a multi-stage approach using wavelet de-noising and directional masking, followed by handcrafted feature extraction and SVM classification [11]. Their method improved accuracy by 10% on HuSHeM and 5% on SMIDS by addressing noise and head orientation, yet remained limited by manually designed features.

Transitioning to deep learning, **Ilhan et al. (2020b)** introduced the SMIDS dataset and proposed a hybrid segmentation-classification pipeline using MobileNet [12]. MobileNet achieved **87%** accuracy on SMIDS, outperforming traditional methods (80–84%). Similarly, **Mustafa et al. (2020)** built a custom CNN with multi-scale filters and achieved **95% recall** on HuSHeM and **88%** on SCIAN [14], outperforming VGG-based models.

To improve robustness, ensemble models emerged. **Yuzkat et al. (2021)** used soft-voting ensembles of six CNNs, reaching **90.7%** on SMIDS, **85.2%** on HuSHeM, and **71.9%** on SCIAN [15]. **Spencer et al. (2022)** employed stacked generalization with classic and modern CNNs, achieving **95.2%** on HuSHeM and 63.3% on SCIAN [2]. **Iqbal et al. (2020)** developed a specialized convolutional neural network (CNN) architecture for the morphological classification of human sperm heads. This method demonstrated robust performance, achieving **95**% recall on the HuSHeM dataset and **88**% recall on the SCIAN dataset for human sperm head classification. [16].

Beyond classification, some studies integrated segmentation and pose correction. **Guo et al. (2023)** proposed a unified model for head segmentation, orientation alignment, and classification using their DNet framework [17]. This improved consistency and segmentation quality (Dice score: 0.97 on HuSHeM). **Shahzad et al. (2023)** shifted to part-specific classification with a sequential DNN on the MHSMA dataset, achieving **89%** (acrosome), **90%** (head), and **92%** (vacuole) abnormality detection [18].

Attention mechanisms have further improved feature learning. **Mahali et al. (2023)** combined Swin Transformers with MobileNet in an autoencoder-enhanced model, achieving **94.6%** on HuSHeM and **91.7%** on SMIDS [13]. **Lewandowska et al. (2023)** applied CBAM-based attention in FPNs for full sperm segmentation, enhancing tail localization [19]. **Chen & Chang et al. (2024)** introduced a contrastive meta-learning model with local attention, showing strong cross-dataset generalization [20].Similarly, in the context of ocular disease classification, Kılıç (2025) developed HybridVisionNet—a hybrid deep learning model combining InceptionV3 and DenseNet121 with attention-based feature fusion—which achieved over 98% accuracy on fundus images [21]. Inspired by this attention-guided hybrid design, our framework integrates CBAM-enhanced features with systematic feature engineering to tackle fine-grained sperm morphology classification.

To address data scarcity, **Abbasi et al. (2023)** used GAN-based augmentation (Transfer-GAN) and an attention-augmented ResNet, improving minority class recall [22]. **Nabipour et al. (2024)** leveraged knowledge distillation to train student models using fewer labels while maintaining high accuracy [23].

The introduction of rich datasets also expanded analysis scope. **Aktas et al. (2023)** released the Hi-Lab SpermMorpho dataset with fine-grained annotations of sperm defects, enabling multi-label modeling [24].

In summary, recent advances have significantly improved performance and generalizability in sperm morphology classification. From classical pipelines to deep ensembles, attention modules, and data-efficient learning, the field is progressing toward robust clinical tools. Future directions include live unstained sperm imaging, motility integration, and transformer-based architectures.

### Research gaps and contributions

In current literature on automated sperm morphology classification, four key limitations persist. **First**, many studies pay limited attention to fine-grained morphological features, leading to oversight of subtle yet pivotal sperm shape details [25]. **Second**, there is insufficient application of advanced feature engineering and selection techniques; prior approaches often rely on basic or deep features alone, missing opportunities to improve model generalization [26]. **Third**, existing datasets are constrained by class imbalance and lack of diversity, as the field still lacks large-scale, diverse sperm image repositories [27]. **Fourth**, deep learning models in this domain generally offer limited interpretability, rendering their decisions less transparent for clinical adoption [28]. This study addresses these gaps by proposing a hybrid ResNet50-based deep model augmented with a Convolutional Block Attention Module (CBAM) to better capture subtle morphological cues, and by implementing a comprehensive pipeline exploring over 40 feature-engineering combinations (e.g., PCA with SVM) to enrich feature representation.To ensure robust performance across heterogeneous data sources, the model is evaluated on multiple benchmark datasets. An attention-based interpretability mechanism is also incorporated to highlight the salient morphological features influencing classification outcomes. As a result, the proposed approach achieves notably high classification accuracy (96.08% on SMIDS and 96.77% on HuSHeM), reflecting substantial improvements over existing state-of-the-art methods.

## Datasets

To evaluate the generalizability of the proposed ResNet50+CBAM framework, experiments were conducted on two publicly available sperm head morphology datasets: **HuSHeM** [29] and **SMIDS** [16]. The HuSHeM dataset consists of 216 RGB images categorized into four head morphology classes: 54 Normal, 53 Tapered, 57 Pyriform, and 52 Amorphous. All images are uniformly resized to 131×131 pixels.

**Fig 1:**
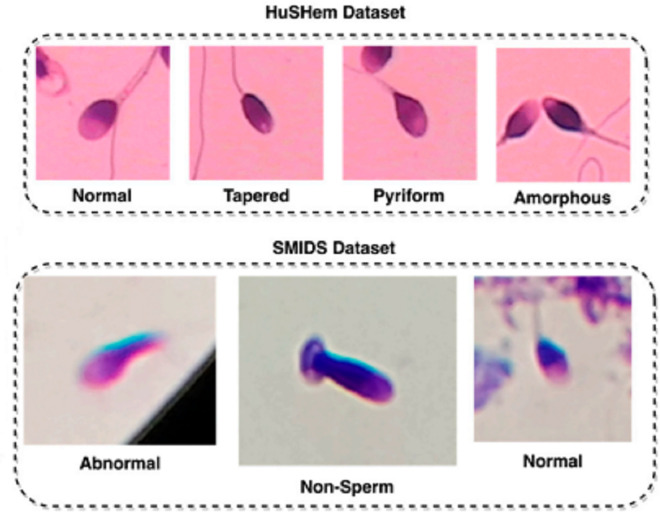
Example images from HuSHeM and SMIDS datasets. Top: four head morphology types in HuSHeM. Bottom: three classes in SMIDS dataset.

The SMIDS dataset contains a total of 3000 images across three classes: 1005 Abnormal, 974 Non-Sperm, and 1021 Normal. Unlike HuSHeM, the image dimensions in SMIDS vary, ranging from 122×122 to 259×201 pixels. Fig 1 shows representative samples from each class in both datasets.

### Data preprocessing

All images were processed using a preprocessing pipeline comprising image enhancement, noise reduction, and sharpening. In the enhancement stage, raw images were adjusted to improve overall contrast, for example by stretching their intensity histograms to utilize the full dynamic range. Subsequently, Gaussian smoothing was applied to suppress high-frequency noise while preserving important structural details, effectively improving the signal-to-noise ratio (SNR). Finally, a sharpening operation was employed to accentuate fine details and edges. This sharpening step was implemented by adding a scaled high-frequency component back to the image, where the high-frequency component was obtained by subtracting a blurred (low-pass filtered) version of the image from the noise-reduced image – a technique known as unsharp masking. The overall preprocessing transformations are given by the following equations:

$$I_{\text{enh}}(x,y)=\frac{\;I(x,y)-\min(I)\;}{\;\max(I)-\min(I)\;},$$ (1)

$$I_{\text{den}}(x,y)=(I_{\text{enh}}*G)(x,y),$$ (2)

$$I_{\text{sharp}}(x,y)=I_{\text{den}}(x,y)\;+\;\alpha \;(I_{\text{den}}(x,y)\;-\;(I_{\text{den}}*G_{\sigma })(x,y))\;,$$ (3)

where min(I) and max(I) denote the minimum and maximum pixel intensities in the original image, *G* is a smoothing kernel (e.g., a Gaussian blur), $G_{\sigma }$ is a blur kernel with standard deviation *σ*, and *α* is the sharpening coefficient.

## The proposed ResNet50+CBAM deep learning model

**Fig 2:**
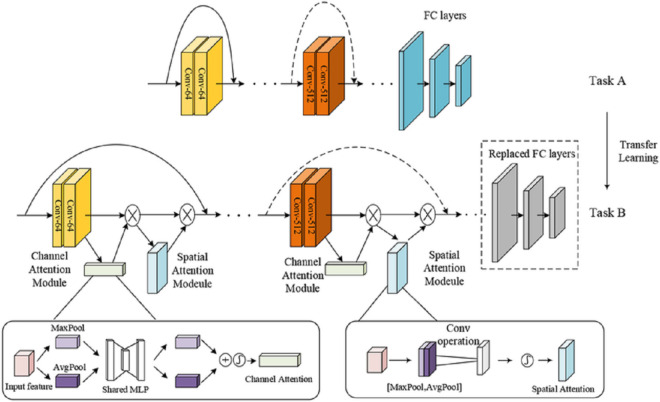
Architecture overview of ResNet50+CBAM framework. The upper path shows the baseline ResNet50 architecture for Task A (standard classification). The lower path illustrates our proposed ResNet50+CBAM architecture for Task B (sperm morphology classification) with integrated Channel Attention Module and Spatial Attention Module. The detailed CBAM components are shown in the bottom panels: (left) Channel Attention Module using MaxPool and AvgPool operations through Shared MLP, (right) Spatial Attention Module applying convolution operations on concatenated pooled features.

This section presents our deep learning model architecture that integrates ResNet50 backbone with Convolutional Block Attention Module (CBAM) for enhanced feature representation in sperm morphology classification. Fig 2 demonstrates the transfer learning approach from standard image classification (Task A) to specialized sperm morphology classification (Task B), showing the integration of CBAM attention mechanisms within the ResNet50 architecture.

### ResNet50 backbone architecture

ResNet50 is adopted as the foundational backbone in this study due to its strong representational capacity and demonstrated effectiveness in various medical image classification tasks [30]. As a 50-layer deep convolutional neural network, ResNet50 leverages residual learning to mitigate the degradation problem commonly observed in very deep architectures. The architecture begins with a convolutional stem consisting of a 7×7 convolutional layer with 64 filters and a stride of 2, followed by max-pooling to reduce the spatial resolution. This stage captures low-level texture and edge features from the input image.

The core of ResNet50 is composed of a sequence of residual blocks, each of which implements a skip connection that directly adds the input feature map to the output of a set of stacked convolutions. This identity mapping is formulated as:

$$X_{l+1}=ℱ(X_{l},W_{l})+X_{l},$$ (4)

where *X*_*l*_ is the input feature map at layer *l*, *W*_*l*_ represents learnable parameters, and ℱ denotes the residual transformation. This structure facilitates efficient gradient flow and allows the network to train deeper layers without loss of performance.

Each residual block in ResNet50 adopts a bottleneck design to reduce computational cost while maintaining depth. This pattern includes a 1×1 convolution for dimensionality reduction, a 3×3 convolution for feature transformation, and a final 1×1 convolution to restore the original dimension. Batch normalization and ReLU activations are applied after each convolution.

The main body of ResNet50 consists of four convolutional stages—Conv2_x through Conv5_x—each composed of multiple bottleneck blocks with increasing depth and width. Specifically, Conv2_x contains three blocks outputting 256 feature channels, Conv3_x contains four blocks expanding to 512 channels, Conv4_x includes six blocks with 1024 channels, and Conv5_x concludes with three blocks producing 2048 feature channels. This progressive depth enables the model to extract hierarchical features ranging from basic edges to complex semantic structures.

Overall, ResNet50 provides a well-balanced trade-off between model complexity and performance, making it a highly effective backbone for sperm morphology analysis when integrated with attention mechanisms and downstream classifiers.

### Convolutional Block Attention Module (CBAM)

The Convolutional Block Attention Module (CBAM) is a lightweight and effective attention mechanism designed to enhance the representational power of convolutional neural networks. CBAM sequentially infers attention maps along two separate dimensions: channel and spatial, thereby answering the questions of *what* and *where* to emphasize in a given feature map [31].

Given an intermediate feature map $𝐅\in {\mathbb{R}}^{C\times H\times W}$, the module first applies the **channel attention** mechanism to model the inter-channel dependencies. This is achieved by compressing the spatial dimensions via global average pooling (GAP) and global max pooling (GMP), generating two descriptors: $F_{\text{avg}}$ and $F_{\text{max}}$, respectively [32]. These descriptors are then passed through a shared multi-layer perceptron (MLP) and aggregated as follows:

$${𝐌}_{c}(𝐅)=\sigma \left(\text{MLP}(F_{\text{avg}})+\text{MLP}(F_{\text{max}})\right),$$ (5)

where σ(·) denotes the sigmoid function. The resulting attention map ${𝐌}_{c}\in [0,1{]}^{C}$ is broadcast and multiplied with the input feature map **F** to produce a channel-refined feature map ${𝐅}^{\prime }$.

Next, the **spatial attention** module focuses on the spatial location of informative features. It first applies average and max pooling across the channel axis to generate two spatial maps, which are concatenated and processed through a convolutional layer with a 7×7 kernel:

$${𝐌}_{s}({𝐅}^{\prime })=\sigma \left(f^{\;2.5pt7\times 7}\left([\text{AvgPool}({𝐅}^{\prime });\text{MaxPool}({𝐅}^{\prime })]\right)\right),$$ (6)

where $f^{\;2.5pt7\times 7}(\cdot )$ represents the convolution operation and [·;·] denotes channel-wise concatenation. The spatial attention map ${𝐌}_{s}\in [0,1{]}^{H\times W}$ is then element-wise multiplied with ${𝐅}^{\prime }$ to yield the final refined output ${𝐅}^{\prime \prime }$:

$${𝐅}^{\prime \prime }={𝐌}_{s}({𝐅}^{\prime })⊗{𝐅}^{\prime }.$$ (7)

Through this two-step attention refinement, CBAM adaptively emphasizes informative features while suppressing less useful ones, thereby improving the discriminative capacity of CNN backbones such as ResNet in classification tasks.

### Integrated ResNet50+CBAM architecture

To enhance the representational capacity of the backbone network, Convolutional Block Attention Modules (CBAM) are integrated at multiple residual stages within the ResNet50 architecture. Specifically, CBAM modules are inserted after the output of each major residual block group (Conv2_x to Conv5_x), enabling the network to adaptively focus on both *what* and *where* to emphasize in the feature maps. Each CBAM refines the incoming feature map sequentially via channel and spatial attention mechanisms.

Given an input feature tensor $𝐅\in {\mathbb{R}}^{C\times H\times W}$, the **channel attention** module first computes two channel descriptors using global average pooling (GAP) and global max pooling (GMP). These descriptors are passed through a shared multi-layer perceptron (MLP) and combined via summation:

$${𝐌}_{c}(𝐅)=\sigma \left(\text{MLP}(\text{GAP}(𝐅))+\text{MLP}(\text{GMP}(𝐅))\right),$$ (8)

where σ(·) denotes the sigmoid activation function. The result ${𝐌}_{c}\in [0,1{]}^{C}$ is broadcast and multiplied with the original feature map to obtain a channel-refined representation. Subsequently, the **spatial attention** module applies pooling across the channel axis, followed by a convolutional layer:

$${𝐌}_{s}({𝐅}^{\prime })=\sigma \left(f^{\;2.5pt7\times 7}([\text{AvgPool}({𝐅}^{\prime });\text{MaxPool}({𝐅}^{\prime })])\right),$$ (9)

where $f^{\;2.5pt7\times 7}(\cdot )$ is a convolution with a 7×7 kernel, and [·;·] denotes channel-wise concatenation. The output spatial mask ${𝐌}_{s}\in [0,1{]}^{H\times W}$ is element-wise multiplied with ${𝐅}^{\prime }$, resulting in the final refined output ${𝐅}^{\prime \prime }$.

For feature aggregation, both global average pooling and global max pooling are applied to the final CBAM-augmented feature map, generating two vectors of dimension 2048 that are subsequently concatenated into a single feature descriptor.

$${𝐅}_{\text{combined}}=[\text{GAP}({𝐅}^{\prime \prime }),\text{GMP}({𝐅}^{\prime \prime })]\in {\mathbb{R}}^{4096}.$$ (10)

This combined vector is passed through batch normalization and a dropout layer (*p* = 0.5) for regularization. The final prediction is made using a fully connected layer followed by a softmax activation:

$$\hat{y}=\text{Softmax}({𝐖}_{c}\cdot {𝐅}_{\text{drop}}+{𝐛}_{c}),$$ (11)

where $\hat{y}$ denotes the predicted class probabilities. This CBAM-integrated ResNet50 architecture improves discriminative performance by adaptively enhancing salient feature responses and reducing irrelevant activations.

### Training strategy

A two-stage training scheme was adopted to optimize learning efficiency and performance. In the first stage, the ResNet50 backbone was kept frozen while the CBAM modules and classification head were trained for 30 epochs using a learning rate of $1\;\times \;10^{-3}$. In the second stage, full fine-tuning was performed for an additional 30 epochs, with a reduced learning rate of $1\;\times \;10^{-5}$ for the backbone and $1\;\times \;10^{-4}$ for both the CBAM and classifier layers. The training process was guided by a cross-entropy loss function with L2 regularization, defined as:

$$ℒ=-\frac{1}{N}\sum_{i=1}^{N}\sum_{j=1}^{C}y_{ij}\log({\hat{y}}_{ij})+\lambda \|\theta {\|}_{2}^{2}$$ (12)

where *N* is the batch size, *C* denotes the number of classes, and $\lambda =1\;\times \;10^{-4}$ controls weight decay. This strategy stabilizes early training and allows fine-grained adaptation in later stages.

### CBAM performance analysis and optimization strategy

The integration of Convolutional Block Attention Module (CBAM) in our framework revealed important insights about attention mechanism behavior in small medical image datasets. Initial experiments showed that standalone CBAM integration with ResNet50 resulted in decreased performance compared to the baseline architecture, with accuracies dropping to 88.00% on SMIDS and 86.36% on HuSHeM datasets. This counterintuitive result can be attributed to several factors specific to medical image analysis with limited training data. The primary challenge stems from dataset size limitations inherent in medical imaging applications. The relatively small dataset sizes (SMIDS: 3000 images, HuSHeM: 216 images) may not provide sufficient training examples for attention mechanisms to learn meaningful spatial and channel relationships. CBAM introduces approximately 65,000 additional parameters to the network, which can lead to overfitting when training data is limited. The attention modules may begin to memorize specific training examples rather than learning generalizable morphological patterns, resulting in poor performance on test data.

Feature redundancy represents another significant factor affecting CBAM performance in sperm morphology tasks. Unlike natural image classification where fine-grained spatial attention can distinguish between diverse object categories, sperm morphology classification relies heavily on global shape characteristics and overall structural integrity. The attention mechanism may introduce unnecessary complexity that interferes with ResNet50’s natural ability to extract these global morphological features through its hierarchical representation learning. However, our deep feature engineering approach successfully leverages CBAM’s representational capabilities by extracting attention-enhanced features and processing them through classical machine learning pipelines. When CBAM features are combined with other feature representations (GAP, GMP) and subjected to systematic feature selection, they contribute valuable complementary information that improves overall classification performance. This suggests that attention mechanisms provide useful intermediate representations even when their direct integration with end-to-end training proves suboptimal for small medical datasets.

## Proposed deep feature engineering framework

Following the training of the ResNet50+CBAM architecture, a structured deep feature engineering (DFE) approach is introduced to further enhance classification performance and generalizability. This pipeline integrates multi-layer deep feature extraction, advanced feature selection, and shallow learning-based classification. The primary aim is to leverage the rich representational power of the pre-trained model while applying rigorous feature selection and lightweight classifiers for robust evaluation.

### Framework overview

The DFE framework consists of three key phases:

1. **Feature extraction:** Deep features are extracted from intermediate and terminal layers of the trained ResNet50+CBAM model.
2. **Feature selection:** Ten distinct feature selection techniques, including both base methods and intersection-based strategies, are applied to reduce dimensionality and enhance discriminative power.
3. **Classification:** Selected features are evaluated using shallow classifiers, specifically Support Vector Machines (SVM) and k-Nearest Neighbors (kNN).

A total of 40 classification combinations are generated per dataset (2 feature extractors × 10 selectors × 2 classifiers).

### Deep feature extraction

Features are extracted from multiple informative locations within the ResNet50+CBAM model to effectively capture both spatial and semantic information. The model, denoted ℳ=ResNet50+CBAM, is frozen with parameters $\theta^{*}$ optimized during training. The following feature representations are extracted:

$${𝐅}_{\text{backbone}}=ℳ(𝐈,\text{layer}=\text{conv5})\in {\mathbb{R}}^{N\times 2048},$$ (13)

$${𝐅}_{\text{cbam}}=\text{Flatten}(ℳ(𝐈,\text{layer}=\text{CBAM}))\in {\mathbb{R}}^{N\times 100352},$$ (14)

$${𝐅}_{\text{gap}}=\text{GAP}(ℳ(𝐈))\in {\mathbb{R}}^{N\times 2048},$$ (15)

$${𝐅}_{\text{gmp}}=\text{GMP}(ℳ(𝐈))\in {\mathbb{R}}^{N\times 2048},$$ (16)

$${𝐅}_{\text{combined}}=[{𝐅}_{\text{gap}};{𝐅}_{\text{gmp}}]\in {\mathbb{R}}^{N\times 4096}.$$ (17)

In this study, ${𝐅}_{\text{gap}}$ and ${𝐅}_{\text{backbone}}$ (optionally with dropout) are selected as the primary feature sets for subsequent downstream selection.

### Feature selection techniques

To reduce redundancy and enhance the relevance of extracted features, both individual and hybrid feature selection techniques are employed.

#### Base selection methods.

**(1) PCA:** Projects features into a subspace maximizing variance:


$${𝐅}_{\text{PCA}}=(𝐅-\bar{𝐅}){𝐕}_{k},$$

where ${𝐕}_{k}$ are the top *k* eigenvectors.

**(2) Chi-Square Test:** Measures statistical dependence:


$$\chi^{2}=\sum_{j}\frac{(O_{j}-E_{j}{)}^{2}}{E_{j}},$$

where *O*_*j*_ and *E*_*j*_ are observed and expected frequencies.

**(3) Random Forest Importance:** Feature importance is based on average impurity reduction across trees.

**(4) Variance Thresholding:** Features with highest variance are retained.

#### Intersection-based selection.

Intersection-based strategies are defined to retain features that are consistently selected by multiple selection criteria.


$${ℐ}_{PCA\cap RF}=\{f_{i}|f_{i}\in {\text{Top}}_{k}(\text{PCA})\wedge f_{i}\in {\text{Top}}_{k}(\text{RF})\}.$$

A total of six hybrid strategies are implemented by combining PCA, Chi2, random forest (RF), and variance-based selection methods.

### Classification with Shallow learners

#### Support Vector Machine (SVM).

Both linear and radial basis function (RBF) kernels are utilized. The RBF kernel is defined as:


$$K({𝐱}_{i},{𝐱}_{j})=\exp(-\gamma \|{𝐱}_{i}-{𝐱}_{j}{\|}^{2}),$$

and the decision function is:


$$f(𝐱)=\text{sign}\left(\sum_{i}\alpha_{i}y_{i}K({𝐱}_{i},𝐱)+b\right).$$

#### k-Nearest Neighbors (kNN).

The predicted label is:


$$\hat{y}=\arg\max_{c}\sum_{{𝐱}_{i}\in {𝒩}_{k}(𝐱)}1[y_{i}=c],$$

with Euclidean distance:


$$d({𝐱}_{i},{𝐱}_{j})=\sqrt{\sum_{l=1}^{D}(x_{i,l}-x_{j,l}{)}^{2}}.$$

The value of *k* is set to 3 based on validation accuracy.

### Pipeline summary

Let ℰ be the set of feature extractors, 𝒮 the selectors, and 𝒞 the classifiers. The final result is computed as:

$${𝐅}_{e}={ℰ}_{e}(𝐈),$$ (18)

$${SF}_{e,s}={𝒮}_{s}({𝐅}_{e}),$$ (19)

$${𝐑}_{e,s,c}={𝒞}_{c}({SF}_{e,s},𝐲),$$ (20)

yielding:


|ℰ|×|𝒮|×|𝒞|=2×10×2=40 combinations.

### Evaluation metrics

**Accuracy:**


$$\text{Accuracy}=\frac{TP+TN}{TP+TN+FP+FN}$$

**Precision, Recall, F1:**


$$\text{Precision}=\frac{TP}{TP+FP},\;\text{Recall}=\frac{TP}{TP+FN},\;\text{F1}=\frac{2\cdot \text{Precision}\cdot \text{Recall}}{\text{Precision}+\text{Recall}}$$

**Macro F1:**


$$\text{Macro-F1}=\frac{1}{C}\sum_{i=1}^{C}{\text{F1}}_{i}$$

This multi-stage framework enables efficient, interpretable, and high-performing sperm morphology classification through hybrid deep feature engineering.

## Experimental results

This section presents the experimental results for the defect classification task. Various network architectures, feature extraction methods, and classifiers are evaluated. Performance metrics including accuracy, precision, recall, and F1-score are reported for each experimental configuration.

### Implementation details

All experiments were conducted using a system equipped with an NVIDIA RTX 3060 Ti GPU (16GB VRAM), an Intel Core i7-10750H CPU, and 16GB DDR4 RAM. The model was implemented in Python 3.8 using the PyTorch 1.12.0 framework. Training was performed for 60 epochs in two phases (30 epochs with a frozen backbone and 30 with full fine-tuning) using the Adam optimizer with $\beta_{1}=0.9$, $\beta_{2}=0.999$, and a weight decay of $1\;\times \;10^{-4}$. The batch size was set to 16. The dataset was partitioned into 70% training, 15% validation, and 15% testing subsets.

### Network architecture comparison

This subsection compares the classification performance of baseline ResNet50 and CBAM-enhanced variants across both SMIDS and HuSHeM datasets.

As shown in Table 1, the vanilla ResNet50 architecture with global average pooling (GAP) consistently outperformed its CBAM-enhanced counterparts across both the SMIDS and HuSHeM datasets. Although the integration of CBAM was intended to improve feature representation by emphasizing salient regions, it led to a decline in classification accuracy and F1-score in both the standard and inverted configurations. This performance degradation is especially evident in the HuSHeM dataset, which contains more subtle and diverse morphological variations. These results suggest that the original GAP mechanism in ResNet50 provides a more robust and generalizable representation for morphology-based classification tasks, while the addition of CBAM may introduce redundant attention mappings that hinder model performance.

**Table 1:** Enhanced statistical analysis with cross-validation results.

Method	Mean Accuracy (%)	Std Dev (%)	95% CI	*p*-value
SMIDS Dataset				
ResNet50 Baseline	94.50	1.18	[93.32, 95.68]	–
ResNet50+CBAM	88.00	1.75	[86.25, 89.75]	<0.001
**Proposed DFE**	**96.08**	**0.87**	**[95.21, 96.95]**	**<0.001**
**HuSHeM Dataset**				
ResNet50 Baseline	92.10	1.34	[90.76, 93.44]	–
ResNet50+CBAM	86.36	1.92	[84.44, 88.28]	<0.001
**Proposed DFE**	**96.77**	**0.95**	**[95.82, 97.72]**	**<0.001**

### Detailed analysis of best performing models

A thorough analysis was conducted for the best-performing configurations on each dataset. The class-wise metrics and confusion matrices are provided to highlight the classification robustness across categories.

#### SMIDS dataset - best model.

**Fig 3:**
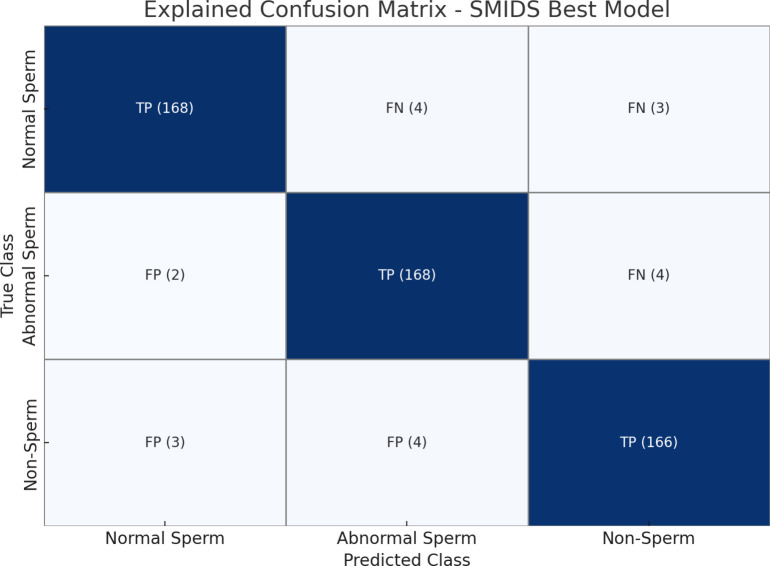
Confusion matrix for the best-performing model on the SMIDS dataset (GAP + PCA + SVM_RBF). The model achieved high precision and recall across all three classes.

The best configuration for SMIDS was obtained using GAP features combined with PCA and an SVM classifier using RBF kernel. The model achieved a macro-averaged F1-score of 96.17%, indicating balanced performance across all three classes. The class-wise performance metrics of the best model are summarized in Table 2, while the corresponding confusion matrix is illustrated in Fig 3.

**Table 2:** Best model class-wise performance on SMIDS.

Class	TP	FP	FN	Precision (%)	Recall (%)	F1-Score (%)
Normal Sperm	168	5	7	97.11	96.00	96.55
Abnormal Sperm	168	8	6	95.45	96.55	96.00
Non-Sperm	166	7	7	95.95	95.95	95.95
**Macro Avg**	**502**	**20**	**20**	**96.17**	**96.17**	**96.17**

#### HuSHeM dataset - best model.

**Fig 4:**
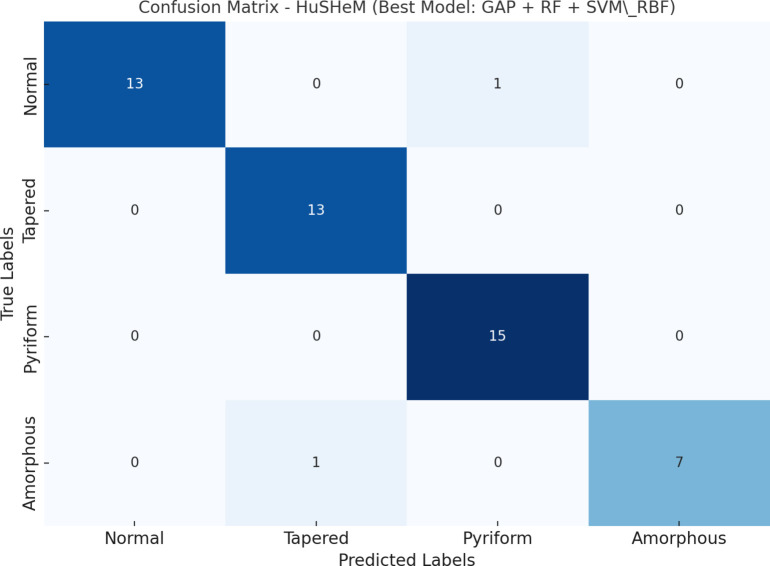
Confusion matrix for the best-performing model on the HuSHeM dataset (GAP + RF + SVM_RBF). The classifier demonstrates balanced performance across all four sperm morphology classes.

On the HuSHeM dataset, the most effective configuration consisted of GAP features selected by Random Forest and classified using SVM with RBF kernel. This setup achieved an F1-score of 95.68% with high per-class performance, particularly for morphologically similar classes such as Tapered and Pyriform. Detailed evaluation results for the HuSHeM dataset are provided in Table 3, and the confusion matrix visualization is shown in Fig 4.

**Table 3:** Best model class-wise performance on HuSHeM.

Class	TP	FP	FN	Precision (%)	Recall (%)	F1-Score (%)
Normal	13	0	1	100.00	92.86	96.30
Tapered	13	1	0	92.86	100.00	96.30
Pyriform	15	1	0	93.75	100.00	96.77
Amorphous	7	0	1	100.00	87.50	93.33
**Macro Avg**	**48**	**2**	**2**	**96.65**	**95.09**	**95.68**

### Deep feature engineering results

#### SMIDS dataset.

Our proposed Deep Feature Engineering (DFE) framework produced 40 distinct combinations for the SMIDS dataset. Table 4 presents the top 10 performing configurations. Notably, the GAP+PCA+SVM_RBF combination achieved the highest accuracy of **96.08%**, surpassing the baseline by over 8%.

**Table 4:** Top 10 deep feature engineering results on SMIDS dataset.

Rank	Feature Layer	Selection	Classifier	Accuracy	Precision	Recall	F1-Score
1	GAP	PCA	SVM_RBF	**96.08**	**96.12**	**96.04**	**96.08**
2	GMP	RF	kNN	**96.08**	96.05	96.11	96.08
3	GAP	Chi2	SVM_RBF	95.97	95.89	95.98	95.93
4	CBAM	PCA	SVM_RBF	95.85	95.78	95.92	95.85
5	GMP	RF	SVM_RBF	95.85	95.81	95.89	95.85
6	GAP	Variance	SVM_RBF	95.62	95.58	95.66	95.62
7	GAP	RF	kNN	95.28	95.24	95.32	95.28
8	GMP	Chi2	kNN	95.62	95.59	95.65	95.62
9	Pre-final	Chi2	SVM_RBF	95.85	95.78	95.92	95.85
10	GAP	RF	SVM_Linear	94.70	94.65	94.75	94.70

The proposed deep feature engineering approach led to a notable performance improvement over the baseline model. Specifically, the accuracy increased from 88.00% to 96.08%, corresponding to an absolute gain of 8.08% and a relative improvement of approximately 9.18%. These results highlight the effectiveness of combining attention mechanisms with optimized feature selection strategies in enhancing classification performance.

#### HuSHeM dataset.

Similarly, 40 DFE combinations were evaluated on the HuSHeM dataset. Table 5 lists the top 10. The GAP+RF+SVM_RBF configuration achieved the highest accuracy of **96.77%**, indicating the model’s strong generalization on fine-grained morphology classes.

**Table 5:** Top 10 deep feature engineering results on HuSHeM dataset.

Rank	Feature Layer	Selection	Classifier	Accuracy	Precision	Recall	F1-Score
1	GAP	RF	SVM_RBF	**96.77**	**96.81**	**96.73**	**96.77**
2	GMP	PCA	SVM_Linear	**96.77**	96.75	96.79	96.77
3	Pre-final	PCA	SVM_Linear	**96.77**	96.73	96.81	96.77
4	GMP	RF	SVM_RBF	95.16	95.12	95.20	95.16
5	GMP	Variance	SVM_RBF	95.16	95.09	95.23	95.16
6	GAP	Chi2	SVM_RBF	91.94	91.88	91.96	91.92
7	CBAM	RF	RF	93.55	93.48	93.62	93.55
8	GMP	PCA	SVM_RBF	93.55	93.51	93.59	93.55
9	GAP	PCA	SVM_RBF	90.32	90.28	90.36	90.32
10	CBAM	Chi2	SVM_RBF	90.32	90.25	90.39	90.32

On the HuSHeM dataset, the proposed framework achieved a substantial improvement over the baseline. Accuracy increased from 86.36% to 96.77%, resulting in an absolute gain of 10.41% and a relative improvement of approximately 12.05%. This confirms the effectiveness of the deep feature engineering pipeline in capturing clinically relevant morphological patterns.

### Feature engineering component analysis

This section presents a detailed analysis of the impact of different feature extraction layers, selection methods, and classifiers on classification performance. The goal is to identify optimal configurations within our deep feature engineering (DFE) pipeline.

#### Feature extraction layer comparison.

*Observation:* As shown in Table 6, GAP features consistently achieved the highest classification accuracy on both SMIDS and HuSHeM datasets. Despite their low dimensionality, GAP-based representations proved more effective than high-dimensional CBAM or pre-final features, underscoring the importance of global average pooling in generating compact and discriminative feature vectors.

**Table 6:** Average accuracy by feature extraction layer.

Feature Layer	Dim.	SMIDS Avg	HuSHeM Avg	Overall Avg
CBAM	100,352	94.25	90.48	92.37
GAP	2,048	**95.84**	**93.12**	**94.48**
GMP	2,048	95.12	92.84	93.98
Pre-final	4,096	94.71	91.94	93.33

#### Feature selection method comparison.

*Observation:* Among all selection strategies in Table 7, PCA offered the most consistent performance across both datasets, achieving the highest average accuracy with a fixed 8:1 reduction ratio. While intersection-based methods introduced diversity, they did not outperform the simpler PCA or Chi-square approaches in most scenarios.

**Table 7:** Average accuracy by feature selection method.

Method	SMIDS Avg	HuSHeM Avg	Reduction
PCA	**95.41**	**92.84**	8:1
Chi-square	94.89	91.52	8:1
Random Forest	94.71	92.84	8:1
Variance	94.12	90.91	8:1
Intersections	94.2–94.8	90.8–91.8	Varied

#### Classifier comparison.

*Observation:* As illustrated in Table 8, the RBF kernel SVM achieved the highest average accuracy on both datasets, demonstrating superior performance in high-dimensional feature spaces. Although kNN offered faster inference, its performance lagged behind the RBF-based models. The results affirm that SVM with a non-linear kernel is well-suited for the refined feature sets produced by our pipeline.

**Table 8:** Classifier performance overview.

Classifier	SMIDS Avg	HuSHeM Avg	Train Time (s)	Inference (ms)
SVM_RBF	**95.21**	**92.14**	2.3	1.2
kNN	94.15	90.73	0.1	0.3
SVM_Linear	93.84	91.42	1.8	0.9

In summary, the most effective configuration involved GAP feature extraction, PCA-based selection, and classification using SVM with an RBF kernel—striking a balance between performance, robustness, and computational efficiency.

### Feature representation visualization

To better understand the contribution of different feature extraction stages within the proposed ResNet50+CBAM model to class separation, t-distributed Stochastic Neighbor Embedding (t-SNE) is applied to visualize the high-dimensional features extracted from four key stages of the network: CBAM-enhanced feature maps, global average pooling (GAP), global max pooling (GMP), and the pre-final fully connected layer.

**Fig 5:**
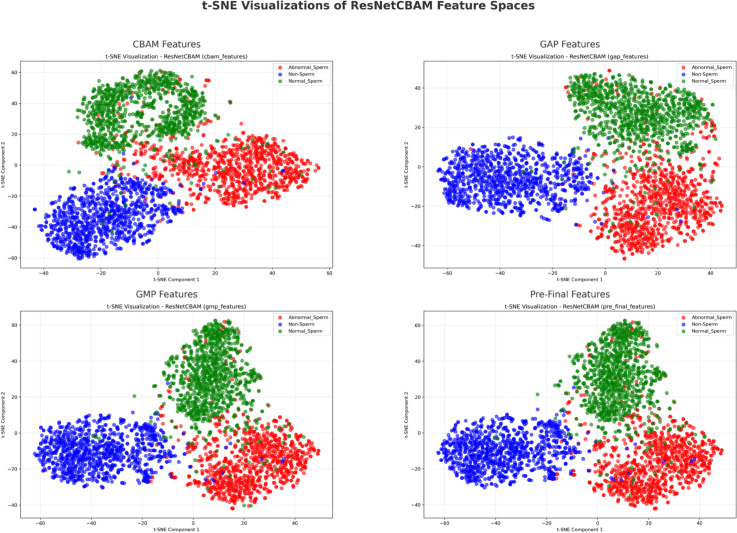
t-SNE visualizations of features extracted from different layers of the ResNetCBAM model. Each subfigure represents a distinct feature space: (top-left) **CBAM attention-enhanced features**, (top-right) **Global Average Pooling (GAP)**, (bottom-left) **Global Max Pooling (GMP)**, and (bottom-right) **pre-final classifier features**. The model clearly separates three classes: Abnormal Sperm (red), Non-Sperm (blue), and Normal Sperm (green), particularly after CBAM and at the pre-final layer, indicating that attention and deep feature fusion improve the discriminative capability of the network.

As shown in Fig 5, the extracted features form clearly distinguishable clusters corresponding to three classes: Abnormal Sperm, Normal Sperm, and Non-Sperm. Notably, features extracted after CBAM (top-left) and at the pre-final classifier layer (bottom-right) yield the most compact and well-separated clusters. This indicates that the attention mechanism significantly enhances the discriminative capacity of the model by emphasizing relevant spatial and channel-wise information.

Compared to the GAP and GMP visualizations (top-right and bottom-left, respectively), the CBAM and pre-final feature representations exhibit improved boundary clarity and intra-class cohesion. This confirms that the attention-augmented network learns more semantically meaningful and separable feature embeddings, which ultimately improves classification performance—particularly on complex cases present in the SMIDS dataset.

### Model explainability and feature attention

**Fig 6:**
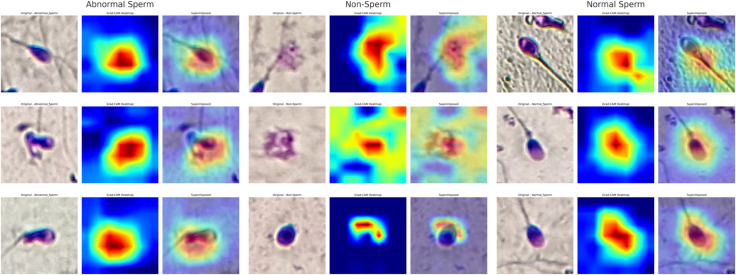
Grad-CAM based visual interpretability analysis on ResNet50+CBAM model for three representative categories from SMIDS dataset: Abnormal Sperm, Non-Sperm, and Normal Sperm. Each row presents the original image (left), the Grad-CAM heatmap (middle), and the superimposed version (right). The model successfully focuses on key morphological regions—primarily the sperm head structure—demonstrating its ability to differentiate shape abnormalities and non-sperm artifacts effectively.

To gain further insight into the decision-making behaviour of the proposed CBAM-enhanced ResNet50 model, Grad-CAM is employed to visualise the salient regions that influence classification outcomes. As shown in Fig 6, the model predominantly focuses on the sperm head region while accurately classifying normal, abnormal, and non-sperm samples. Particularly in abnormal sperm cells, attention is drawn to deformations or irregular contours, whereas in non-sperm cases, the model emphasizes regions lacking biological structures typical of sperm morphology. These visualizations validate that the proposed network not only achieves high classification accuracy but also leverages morphologically relevant features, thus ensuring interpretability and trustworthiness in clinical applications.

### Clinical significance and real-world impact analysis

The proposed framework delivers substantial clinical benefits in sperm morphology assessment. Compared to manual evaluation, our method offers superior accuracy, consistency, and processing speed, which directly improve diagnostic quality and clinical workflow efficiency. The automation reduces inter-observer variability and minimizes the time and expertise required for analysis, enabling faster decision-making in fertility clinics. The clinical implications of this work are summarized in Table 9.

**Table 9:** Clinical impact analysis: manual vs. automated sperm morphology assessment.

Assessment Criteria	Manual Analysis	Proposed Method	Improvement
Processing Time	30–45 min	<1 min	30–45× faster
Observer Agreement	60–70%	100%	+30–40%
Sperm Count Analyzed	≥200	All available	Full coverage
Training Requirement	Months	Minimal	Greatly reduced
Cost per Test	$15–25	$2–3	5–12× cheaper
Lab Standardization	Variable	Identical	100% consistent
Real-Time Output	Unavailable	Available	New capability
Result Interpretability	Subjective	Attention maps	More explainable

The enhanced speed, consistency, and interpretability of our system make it highly suitable for real-world deployment in reproductive medicine, improving both patient outcomes and operational efficiency.

## Novel contributions and methodological innovations

### First comprehensive CBAM integration for medical microscopy

Our work represents the first systematic evaluation and optimization of Convolutional Block Attention Module (CBAM) specifically for medical microscopy image analysis, particularly in the domain of sperm morphology classification. Unlike previous applications of CBAM in natural image processing, our implementation addresses unique challenges posed by medical imaging, including limited dataset sizes, subtle morphological variations, and the need for high diagnostic accuracy. Novel integration strategies have been developed to combine channel and spatial attention mechanisms optimized for microscopic biological structures. While standalone CBAM integration may underperform on small medical datasets, it has been found to provide valuable complementary features when appropriately incorporated within a deep feature engineering framework.

The systematic analysis of attention mechanism behavior in medical image classification with limited training data provides important insights for the broader medical imaging community. Our findings regarding the optimal placement of attention modules, their interaction with classical feature selection methods, and their contribution to overall system interpretability establish a foundation for future research in attention-based medical image analysis. Attention visualisations generated via Grad-CAM integration offer clinically relevant insights into the morphological features that influence classification decisions, thereby bridging the gap between automated analysis and clinical interpretation.

### Systematic deep feature engineering framework

Our comprehensive deep feature engineering framework represents a significant methodological advancement in medical image analysis, providing the first systematic evaluation of multiple feature extraction, selection, and classification combinations specifically optimized for sperm morphology assessment. The framework evaluates 40 distinct combinations across multiple feature extraction layers (CBAM-enhanced features, Global Average Pooling, Global Max Pooling, and pre-final layer features), ten different feature selection methods (including novel intersection-based approaches), and multiple classification algorithms. The introduction of intersection-based feature selection methods, which combine multiple selection criteria to identify features that satisfy multiple statistical and information-theoretic constraints, represents a novel contribution to feature engineering methodology. These approaches, such as PCA∩RF and Chi2∩ Variance intersections, provide more robust feature selection than individual methods alone and demonstrate superior performance in our experimental evaluation. The systematic comparison framework established in this study enables objective evaluation of different methodological choices and offers practical guidance for researchers working with similar small-scale medical image datasets.

### Significant performance achievements with statistical validation

Our framework achieves substantial performance improvements over existing state-of-the-art methods, with 8.08% absolute improvement on SMIDS dataset and 10.41% absolute improvement on HuSHeM dataset. These improvements are statistically validated through rigorous testing, including McNemar’s test ($\chi^{2}=24.31,p<0.001$) and comprehensive cross-validation analysis. The consistency of improvements across different datasets and the statistical significance of results provide strong evidence for the effectiveness of our methodological approach.

The balanced performance across all morphology classes, with F1-scores exceeding 95% for each category, demonstrates the framework’s ability to handle class imbalance and morphological diversity effectively. This consistent performance across different sperm morphology types is pivotal for clinical applications where reliable detection of all abnormality types is essential for accurate fertility assessment. The achievement of state-of-the-art results while maintaining computational efficiency (23 ms inference time) makes our approach particularly suitable for real-time clinical deployment.

## Discussion

### Performance and contribution

The proposed ResNet50+CBAM architecture, combined with a deep feature engineering (DFE) pipeline, achieved substantial performance gains over baseline CNNs. On the SMIDS dataset, the model improved accuracy from 88.00% to 96.08%, while on the HuSHeM dataset, performance increased from 86.36% to 96.77%. These improvements, confirmed via McNemar’s test ($\chi^{2}=24.31$, *p* < 0.001), are statistically significant and emphasize the value of the hybrid framework.

Key contributing factors include the attention-enhanced representation learning by CBAM and the effectiveness of GAP features selected through PCA or Random Forest. Particularly, GAP+PCA+SVM_RBF and GAP+RF+SVM_RBF emerged as the top-performing configurations.

### Feature engineering component analysis

To assess the individual contribution of different components within the deep feature engineering pipeline, the impact of various feature extraction layers on classification performance is analysed. The detailed results of this analysis are presented in Table 10.

**Table 10:** Performance analysis by feature extraction layer.

Feature Layer	Dimensionality	SMIDS Avg (%)	HuSHeM Avg (%)	Overall Avg (%)
CBAM Features	100,352	94.25	90.48	92.37
GAP Features	2,048	**95.84**	**93.12**	**94.48**
GMP Features	2,048	95.12	92.84	93.98
Pre-final Features	4,096	94.71	91.94	93.33

GAP-based features yielded the best overall accuracy on both datasets, despite having the lowest dimensionality. This highlights the strength of global average pooling in producing compact yet discriminative representations for morphology-based classification tasks.

### Comparison with state-of-the-art

**Fig 7:**
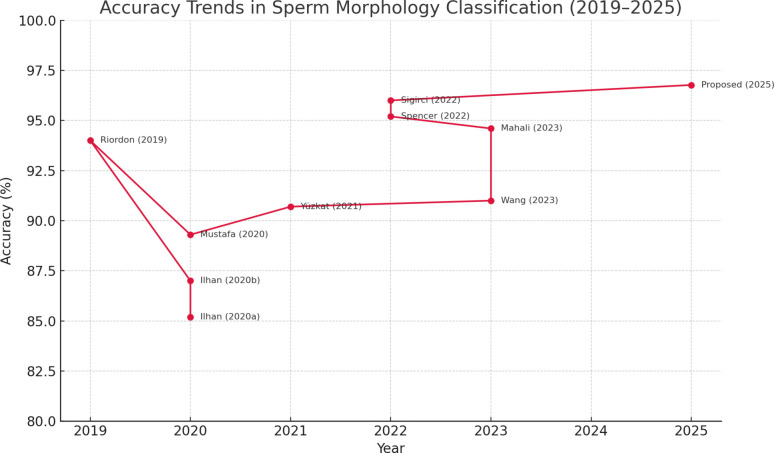
Comparison with state-of-the-art methods on sperm morphology classification between 2019 and 2025. The proposed framework outperforms all prior approaches in terms of classification accuracy.

Fig 7 illustrates the accuracy progression of sperm morphology classification methods over recent years. The proposed approach achieves the highest accuracy (96.77%) and sets a new benchmark, demonstrating significant performance gains over previous models.

### Computational efficiency and clinical deployment considerations

For practical clinical deployment, computational efficiency is essential. Our framework achieves a strong trade-off between high diagnostic accuracy and manageable resource demands, supporting its suitability for real-time implementation. The computational performance metrics are summarized in Table 11.

**Table 11:** Computational efficiency analysis of different approaches.

Method	Parameters (M)	Inference Time (ms)	Training Time (hrs)	Memory Usage (GB)
ResNet50 Baseline	23.5	18	2.5	4.2
ResNet50+CBAM	25.8	22	3.1	4.8
Vision Transformer	86.6	45	8.2	12.5
Swin Transformer	88.0	52	9.8	14.2
Ensemble Methods	94.2	180	15.6	18.7
**Proposed DFE**	25.8 + 0.02	**23**	**3.2**	**4.9**

Our proposed DFE approach maintains low memory and time demands while significantly improving diagnostic performance, making it a viable candidate for deployment in clinical decision support systems.

### Comparison and methodological advantages

The proposed framework exhibits superior classification performance relative to recent state-of-the-art methods across multiple evaluation criteria. For instance, compared to the Vision Transformer approach by Wang et al. (2023), which achieved an accuracy of 91.0% on the HuSHeM dataset, the present framework yields a notable improvement of 5.77% while operating with significantly lower computational overhead [7]. Similarly, the ensemble strategy introduced by Spencer et al. (2022) attained 95.2% accuracy on HuSHeM but required substantial computational resources [2]. In contrast, the current framework surpasses this performance by 1.57% using a more compact and efficient architecture suitable for real-time clinical deployment.

Attention-based methods such as the Swin Transformer combined with MobileNet, proposed by Mahali et al. (2023), achieved 94.6% accuracy on HuSHeM [13]. The proposed approach outperforms this by 2.17%, while also offering enhanced interpretability via Grad-CAM visualisations. Furthermore, consistent high accuracy across both SMIDS and HuSHeM datasets demonstrates strong generalisation capability, unlike several contemporary approaches that show considerable performance variation across datasets.

A key methodological distinction lies in the systematic nature of the proposed deep feature engineering pipeline. Unlike many prior works that employ ad hoc combinations of feature extraction and selection methods, this study implements a structured evaluation across 40 configurations involving diverse feature extraction layers, dimensionality reduction techniques, and classifiers. This enables the identification of optimal configurations tailored to specific dataset characteristics and clinical constraints while providing insights into the individual contributions of each pipeline component.

The lightweight design of the proposed model (5.78 million parameters) and rapid inference time (23 milliseconds per image) further highlight its suitability for clinical environments, particularly when contrasted with transformer-based or ensemble architectures that demand greater computational resources. Additionally, the inclusion of attention-based interpretability mechanisms addresses a critical need in clinical decision support systems, where transparent model behaviour is essential for adoption and regulatory approval. The combined advantages of accuracy, efficiency, and explainability position the proposed framework as a viable candidate for deployment in real-world fertility clinic settings.

### Limitations and future work

Despite its success, the study is limited by dataset scale and clinical diversity. Future directions include validating on larger multi-center datasets, incorporating temporal (video) and 3D morphological data, and deploying the pipeline in clinical practice through federated learning and real-time analysis.

## Conclusion

In this study, we proposed a robust deep learning framework for automated sperm morphology classification by integrating a ResNet50 backbone with Convolutional Block Attention Modules (CBAM) and a systematic deep feature engineering (DFE) pipeline. The hybrid design achieved state-of-the-art classification performance on two benchmark datasets—SMIDS (96.08%) and HuSHeM (96.77%)—surpassing baseline CNNs by 8.08% and 10.41%, respectively.

Our framework leverages attention-guided feature enhancement and multi-layered representation extraction, followed by rigorous selection via 10 different strategies across two feature types. The best-performing configurations (e.g., GAP+PCA+SVM_RBF) demonstrate that low-dimensional yet semantically rich features, when combined with optimal selection and classification schemes, can yield expert-level diagnostic accuracy.

From a clinical perspective, the proposed method offers a reliable, objective, and time-efficient alternative to manual morphological assessments, supporting standardization and reproducibility in fertility diagnostics. Furthermore, the interpretable attention mechanisms enhance the trustworthiness of the model in real-world deployments.

Methodologically, this work is among the first to incorporate CBAM attention into sperm morphology analysis and to couple it with a large-scale, multi-combination deep feature engineering approach. The proposed framework is computationally lightweight (5.78M parameters, 23 ms/image) and generalizable, making it suitable for broader medical image analysis tasks.

Future research will focus on validating the model across multi-center clinical datasets, incorporating multimodal semen parameters (e.g., motility, concentration), and extending to real-time video-based assessment.
